# Effects of Different Degrees of Extraluminal Compression on Hemodynamics in a Prominent Transverse-Sigmoid Sinus Junction

**DOI:** 10.3389/fnhum.2022.823455

**Published:** 2022-02-16

**Authors:** Xiaoyu Qiu, Pengfei Zhao, Zhenxia Mu, Chihang Dai, Xiaoshuai Li, Ning Xu, Heyu Ding, Shusheng Gong, Zhenghan Yang, Bin Gao, Zhenchang Wang

**Affiliations:** ^1^Department of Radiology, Beijing Friendship Hospital, Capital Medical University, Beijing, China; ^2^Faculty of Environment and Life, Beijing University of Technology, Beijing, China; ^3^Department of Otolaryngology Head and Neck Surgery, Beijing Friendship Hospital, Capital Medical University, Beijing, China

**Keywords:** pulsatile tinnitus, cranial venous sinuses, compression degree, computational fluid dynamics (CFD), hemodynamics

## Abstract

**Objectives:**

To simulate hemodynamic changes after extraluminal compression in pulsatile tinnitus (PT) patients with a prominent transverse-sigmoid sinus junction (PTSJ).

**Methods:**

One patient-specific case was reconstructed based on computed tomography venography (CTV) images of a PT patient. The compression degree served as a new index in this study. Cases with 10, 20, 30, 40, 50, 60, 70, 80, and 90% of the compression degree of the control subject were constructed. Steady-state computational fluid dynamics (CFD) were assessed. The wall pressure distribution, wall maximum pressure (P_*max*_) and flow pattern (velocity streamlines and velocity vector) of the PTSJ were calculated to evaluate hemodynamic differences among all cases.

**Results:**

With increasing compression, the wall pressure at the compression point and downstream of the PTSJ decreased but increased upstream. When the compression degree exceeded 70%, the upstream pressure increased significantly. Above 50% compression, the blood flow pattern downstream of the sigmoid sinus tended to spiral, especially after 80% compression. Beyond 60% compression, the blood flow pattern under the compression axis became more medial.

**Conclusion:**

Mechanical compression of PTSJ changes wall pressure and blood flow patterns. The degree of compression should be carefully observed to avoid possible complications or reoccurrence.

## Introduction

Pulsatile tinnitus (PT) is a kind of rhythmic, conscious or even objective noise that is often transmitted to the ear through the temporal bone or vascular structures; the frequency is mostly consistent with the heartbeat. The noise often occurs in childbearing women and has varying degrees of negative impact on the work and life of patients, resulting in insomnia, irritability, anxiety, depression and even suicide. PT can be classified as arterial or venous according to the vascular origin, and venous origin is much more common (Dong et al., 2015; Han et al., 2017). Venous PT can be reduced or eliminated by compression on the affected side of the neck. There are many known causes of venous PT, including ipsilateral venous outflow dominance, transverse sinus stenosis, sigmoid sinus abnormalities (diverticulum, wall dehiscence, and enlargement), emissary vein enlargement, and high jugular bulb localization (Krishnan et al., 2006; Han et al., 2017; Zhao et al., 2020). According to the above different causes, the commonly used surgical methods include stent placement in stenosis, diverticulum restoration, bone wall reconstruction, vein ligation, and so on.

Although previous studies (Guo and Wang, 2015; Hsieh and Wang, 2020) have suggested that a prominent transverse-sigmoid sinus junction (PTSJ) is one of the causes of PT, related reports are limited. In recent years, it has been reported that extraluminal sigmoid sinus angioplasty (ESSA) was used to resolve PT caused by a PTSJ (Wee et al., 2012; Guo and Wang, 2015; Kim et al., 2016), and computational fluid dynamics (CFD) was used to simulate the changes in hemodynamics before and after surgery (Hsieh and Wang, 2020). Although the prognosis is good after this surgical treatment, the compression degree on the enlargement area is determined by the subjective feeling of patients under local anesthesia, and no objective studies on the influence of different compression degrees on the hemodynamics of the PTSJ have been reported to date. Therefore, for PT caused by a PTSJ, relevant research on hemodynamic changes with different surgical compression degrees at this stage is lacking.

In this study, based on the images of one PT case caused by a PTSJ, we used the finite element method to construct models with different compression degrees to perform CFD simulation and analyze the hemodynamic changes in the PTSJ. The inlet velocity was set according to the real blood flow velocity measured by 4D Flow magnetic resonance (MR). To qualitatively and quantitatively evaluate the hemodynamic differences in the enlargement area with different compression degrees, the vessel wall pressure distribution, maximum wall pressure (P_*max*_) and blood flow pattern (velocity streamlines and velocity vectors) were calculated. The aim was to provide a basis for the degree of ESSA.

## Materials and Methods

### Patient Information and Study Design

**FIGURE 1:**
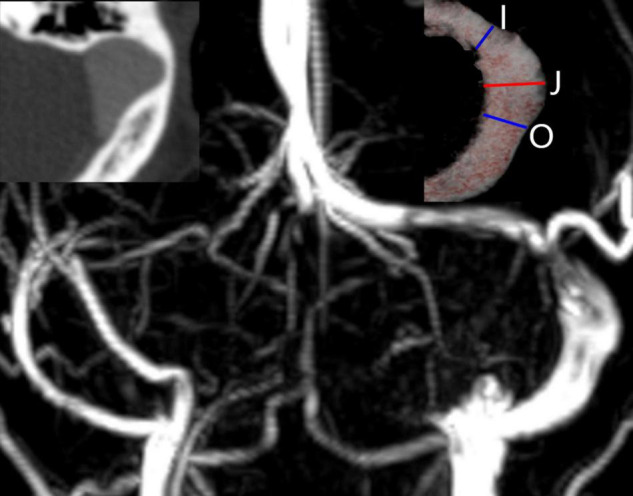
Imaging features of a prominent transverse-sigmoid sinus junction (PTSJ). The PTSJ is located between the blue lines indicating inlet flow (I) and outlet flow (O). The red line (J) indicates the largest PTSJ area.

The medical data used in this study were obtained from our institution and were provided with the patient’s consent. This study was based on a 29-year-old female who presented with a 2-year history of left-sided PT. The noise could be eliminated by compressing the ipsilateral jugular vein. The otoscopic and audiometric evaluations were normal. Computed tomography venography (CTV) and magnetic resonance venography (MRV) revealed ipsilateral venous outflow dominance and a PTSJ. The definition of PTSJ (Figure 1) was based on a study by Hsieh and Wang (2020).

### Imaging Features

Computed tomography venography images were obtained by a 256-slice spiral CT scanner (Philips Medical Systems, Netherlands), and the CTV data consisted of 231 slices (512 × 512 pixels; slice thickness, 0.625 mm) in Digital Imaging and Communications in Medicine format. Iodinated non-ionic contrast material was applied to display the lumen of the transverse sinus to the jugular vein on CTV images. The patient underwent MRV and 4D Flow scans using a 3.0 T MR scanner (Philips, Ingenia, Netherlands). All visualization, assessment and interpretation of 4D Flow data was performed using dedicated GT Flow 2.2.15 software (GyroTools, Switzerland).

### Vascular Model Construction

A preliminary 3D geometric vessel model was reconstructed using Mimics 20.0 software (Materialise, Belgium), including the left transverse sinus, sigmoid sinus and jugular vein. The processed case was imported into Freeform and Geomagic Studio software (Geomagic, North Carolina, United States) for smoothing, and the obtained model was taken as the control subject. The enlargement depth was measured using SolidWorks 2018 software (Dassault systems, Paris, France). The depth was defined as the distance from the highest point on the lateral section of the PTSJ to the lowest point on the inner section. All models with different compression degrees were assembled by SolidWorks. Based on the control subject, the simulated exposed surgical field was established referring to research by Hsieh and Wang (2020), when drawing an ellipse with a short axis and a long axis of 10 and 15 mm, respectively, perpendicular to the enlargement depth. The simulated degree of surgical compression was quantified by depth. With the enlargement depth as the reference line, nine groups of models with different compression degrees from 10 to 90% in increments of 10% were set along the line. The names of cases with different compression degrees and the depth of blood vessels after compression are shown in Table 1.

**TABLE 1:** Cases with different compression degrees and the depth of blood vessels after compression.

Case number	Compression degree (%)	Depth of PTSJ (mm)
Control	0	15
1	10	13.5
2	20	12
3	30	10.5
4	40	9
5	50	7.5
6	60	6
7	70	4.5
8	80	3
9	90	1.5

*PTSJ, prominent transverse-sigmoid sinus junction.*

### Computational Models and Simulations

**FIGURE 2:**
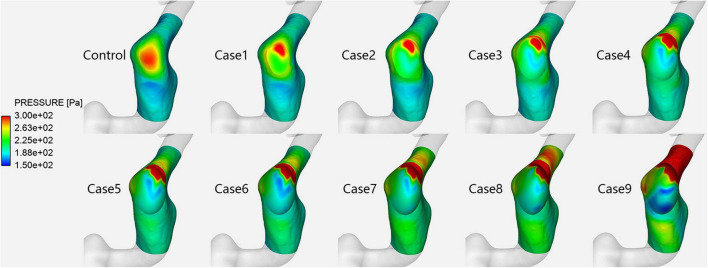
Vessel wall pressure distribution in each case at the prominent transverse-sigmoid sinus junction (PTSJ).

All steps in this section were completed by using the components of ANSYS 2020 R1 software (ANSYS, Inc., Cecil Township, PA, United States). The standard tessellation language format models from SolidWorks were imported into Workbench for Boolean subtraction modeling and then imported into Fluent for meshing, allowing adaptive polyhedral 3D meshing of high quality. Three boundary layers were generated to resolve the flow field at the fluid-wall interface. To confirm the applicable grid size, the average wall pressure of the PTSJ (colored area in Figure 2) was used as the criterion for the grid-independence test. An error of less than 5% was acceptable. A steady-state CFD calculation was performed, the boundary condition of the inlet was set at 0.30 m/s, which was based on the real blood flow velocity measured by 4D Flow MR, and the boundary condition of the outlet was set at 0 absolute pressure. A total of 877,448 elements were developed, and they were sufficient for this study. The maximum element size of each case was set to 0.3 mm. The vessel wall was assumed to be rigid, with no-slip conditions. The blood was assumed to be a laminar, isotropic, homogeneous and incompressible Newtonian fluid with a viscosity of 0.0035 Pa/s and density of 1,050 kg/m^3^. The conservation of mass and momentum for the Navier-Stokes equation, solved with Fluent, was used as the governing equation for calculations. The convergence precision was set at 10^–3^. Based on the CFD simulation results, several flow parameters were calculated and colored by Ensight according to distribution and magnitude to evaluate the effects of model hemodynamics quantitatively. These parameters included the wall pressure distribution, P_*max*_, average wall pressure (P_*avg*_), flow streamlines, velocity vector, maximum velocity (V_*max*_) and average velocity (V_*avg*_).

## Results

### Vessel Wall Pressure

**FIGURE 3:**
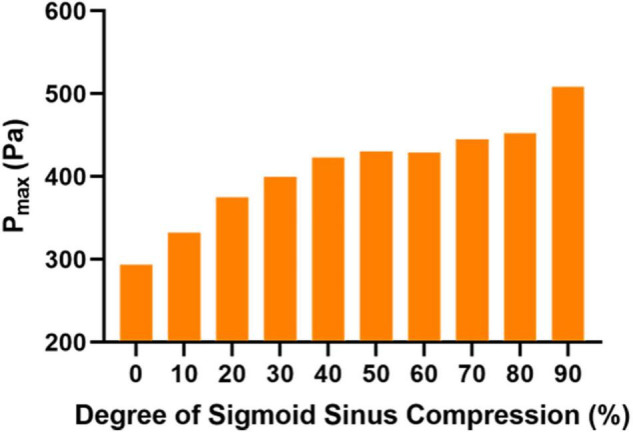
Maximum wall pressure (P_*max*)_ in each case at the prominent transverse-sigmoid sinus junction (PTSJ).

Figure 2 shows the vessel wall pressure distribution at the PTSJ. In the control subject, the wall pressure of the PTSJ was greater than that of the surrounding area. This finding is consistent with previous studies and suggested that increased vessel wall pressure at the PTSJ was a significant cause of PT (Huang et al., 2020; Mu et al., 2020). With increasing compression degree, the wall pressure at the compression point and downstream of the PTSJ decreased, but the wall pressure upstream of the PTSJ slightly increased. The quantitative results (Figure 3 and Table 2) clearly show that the wall pressure changed non-linearly with increasing compression. In addition, Figure 2 shows that when the compression degree reached 70%, the wall pressure increased prominently, especially at the upstream transverse sinus side.

**TABLE 2:** Quantitative values of wall pressure and velocity of the PTSJ with different compression degrees.

Case	P_*max*_ (Pa)	P_*avg*_ (Pa)	V_*max*_ (m/s)	V_*avg*_ (m/s)
Control	293.7	191.5	0.45	0.13
1	332.5	194.6	0.53	0.14
2	375.0	193.9	0.54	0.15
3	400.0	195.8	0.52	0.15
4	422.9	200.4	0.50	0.16
5	430.7	203.6	0.49	0.17
6	429.1	204.7	0.46	0.18
7	445.0	215.8	0.46	0.19
8	452.6	224.0	0.51	0.23
9	508.7	242.5	0.62	0.30

*P_max_, wall maximum pressure; P_avg_, wall average pressure; V_max_, maximum velocity; V_avg_, average velocity.*

### Blood Flow Pattern

**FIGURE 4:**
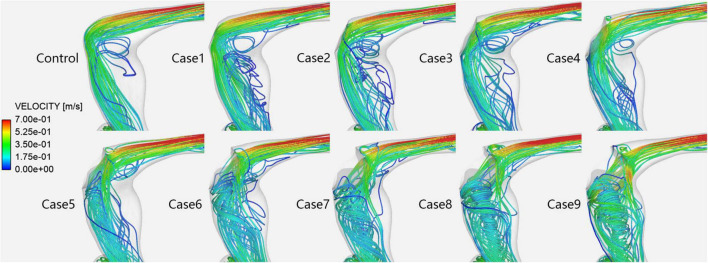
Velocity streamlines in each case at the prominent transverse-sigmoid sinus junction (PTSJ).

Figure 4 shows the velocity streamlines of the PTSJ. The streamlines in cases 1–4 were similar to those in the control subjects. When the compression degree exceeded 50%, the blood through the compression area showed eccentric flow, which led to the gradual transition of blood flow downstream of the compression area from laminar to spiral, especially when the compression degree reached 80%.

**FIGURE 5:**
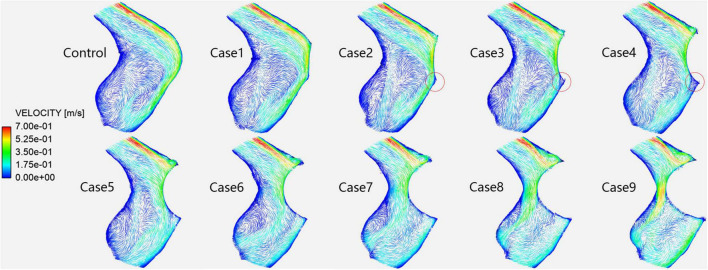
Velocity vectors in each case at the prominent transverse-sigmoid sinus junction (PTSJ). The red circles represent the blood stasis area.

Figure 5 shows the velocity vectors of the PTSJ. After the compression degree increased to 60%, the high-speed blood flow in the lateral area downstream of the compression site gradually moved toward the medial region, the lateral blood flow pattern was more turbulent than before, and the overall blood flow velocity downstream was faster. The quantitative results are shown in Table 2. In addition, there was a sharply defined low-velocity blood flow area immediately below the compression site in cases 2–4, causing local blood flow stasis.

## Discussion

A PTSJ is known as the most vulnerable site for venous PT (Guo and Wang, 2015; Kim et al., 2016). Because of the sharp angle between the transverse sinus and sigmoid sinus (Xue et al., 2012; Kim et al., 2016; Zhao et al., 2016; Huang et al., 2020; Mu et al., 2020), local irregular blood flow and increased wall pressure easily occur. This blood flow impacts and erodes the adjacent temporal bone, resulting in sigmoid sinus wall dehiscence and subsequently producing noise. Therefore, it is important to observe the hemodynamic changes in blood flow in this position. The literature (Cho et al., 2012; Wee et al., 2012; Kim et al., 2016) indicates that high venous blood perfusion is one of the causes of PT. Considering that the blood flow is proportional to the fourth power of the radius (Poiseuille’s law), when the asymmetry between the bilateral venous sinuses is significant, the dominant side may receive almost all the venous blood flow (Roos, 1962). This feature may lead to slow progressive erosion of the bony wall adjacent to the PTSJ (Cho et al., 2012), and the diameter of the PTSJ may gradually increase (Kim et al., 2016). Therefore, in this study, we selected a PT patient with a PTSJ on the affected side and the dominant side of venous sinus returns to construct relevant models for CFD simulation analysis.

The effect and hemodynamic changes of ESSA on PT induced by a PTSJ have been confirmed by relevant studies (Wee et al., 2012; Guo and Wang, 2015; Kim et al., 2016; Hsieh and Wang, 2020). However, this study is the first to objectively simulate the effect of different degrees of surgical compression on the hemodynamics of the PTSJ. In this study, 10 finite element models with different compression degrees were constructed for CFD simulation and analysis. We found that the hemodynamics of the PTSJ were significantly affected by different degrees of surgical compression. With increasing compression, the wall pressure upstream of the compression site increased non-linearly. When the compression degree was greater than 70%, the wall pressure at the PTSJ increased significantly. Moreover, with increasing compression, the blood flow velocity accelerated downstream of the compression site, and the blood flow pattern gradually changed into a spiral shape, significantly changing after the degree of compression reached 80%. These results show that the hemodynamic changes at the PTSJ are significant when the surgical compression degree is large, so the compression degree should be carefully planned.

The vessel wall pressure is the most representative hemodynamic parameter of PT. The PTSJ is known as the most vulnerable site for venous PT. Previous studies (Tian et al., 2017; Mu et al., 2020) have shown that increased wall pressure at the PTSJ may lead to the occurrence or recurrence of sigmoid sinus wall dehiscence and PT. The results of our study show that the pressure at the compression site decreased, which is consistent with the findings of Hsieh and Wang’s (2020) study, which demonstrated that venous sinus compression at the site of wall dehiscence can reduce the pressure and eliminate PT. However, when the degree of compression reached 70%, the pressure upstream of the compression site increased significantly. It is hypothesized that this increase may be related to the different compression methods and other factors, such as irregular compression morphology, inaccurate compression depth and orientation in the actual operation. Therefore, from the perspective of intravascular hemodynamics, it can be inferred that excessive compression is not only not conducive to the elimination of PT but also leads to a wider range of wall dehiscence in the long term. In addition, the mechanism of excessive compression is similar to that of vein ligation and may increase the risk of other complications, such as increased intracranial pressure, contralateral PT and neurological symptoms (Jackler et al., 2001). The compression degree in a previous study (Guo and Wang, 2015) mostly ranged from 46–69% and did not exceed 70%, with a good curative effect, which is indirectly consistent with the results of this study. These findings suggest that when choosing ESSA, the degree of compression should not be excessive (70%) to avoid adverse effects on the veins themselves and related complications.

It has also been considered that turbulence in blood vessels is one of the important causes of PT (Tian et al., 2017; Hsieh and Wang, 2020). In this study, the lateral blood flow of the PTSJ in the control subject was stable laminar flow, which is not involved in the pathogenesis of PT. This finding is different from that reported by Hsieh and Wang (2020), which showed a local turbulence pattern. This difference may be related to the different shapes and expansion degrees of the PTSJ. When the compression degree was less than 50%, the blood flow pattern was almost unchanged. This may be indirectly consistent with the literature (Guo and Wang, 2015), which indicates that the therapeutic effect on PT is the best when the degree of compression is 46–69% and that a patient with a compression degree of only 30% experiences no relief. However, spiral blood flow gradually appeared downstream of the sigmoid sinus when the degree of compression continued to increase, especially when the degree of compression reached 80%. After the normal laminar flow transforms into a spiral blood flow, the blood flow pattern, direction and velocity change, which affects the vascular wall and stimulates vascular endothelial remodeling (Kruger-Genge et al., 2019). In the long run, this may cause PT recurrence or other vascular diseases. Therefore, we should avoid long-term injury caused by excessive compression. In addition, our results also revealed an interesting phenomenon; when the degree of compression was 20–40%, there was a sharp, low-speed, blood stasis area below the compression site, potentially leading to long-term thrombosis. Although real compression does not produce an absolutely sharp edge in the area of compression, there are still similar slightly smooth areas. Thus, this condition plays a certain role in clinical practice.

There are some limitations to this study. First, all models are based on one special patient. Although this approach cannot fully represent the characteristic changes of related cases, the construction of the case morphology and the selection of the boundary conditions are consistent with the real situation. The sample size should be increased for further verification in future work. Second, this study evaluated the effect of simulated surgery, and there was a certain gap with the real surgical state. In follow-up work, the simulation process will be optimized to be as close as possible to the actual situation. Third, the setting of boundary conditions in CFD simulation affects the results, but it does not hinder the horizontal comparison in our research. Final, although venous wall pressure and spiral flow have an important impact on PT, they cannot be used as direct evidence for the elimination or aggravation of PT. We will conduct multi physical field coupling analysis in subsequent research.

## Conclusion

Different degrees of surgical compression produce different pressure fields and flow fields closely related to PT. In this study, although there was no unified threshold to simulate the effects of different compression degrees on the characteristics of vascular wall pressure, blood flow pattern and velocity, it was found that when the compression degree increased, the hemodynamics of the PTSJ exhibited significant abnormal changes. Excessive upstream wall pressure, accelerated blood flow velocity and spiral blood flow instead of laminar flow cause immediate or long-term effects on blood vessels, potentially leading to surgical failure or PT recurrence. Therefore, for PT patients with a PTSJ, it is necessary to avoid excessive compression when deciding to perform ESSA.

## Data Availability Statement

The original contributions presented in the study are included in the article/supplementary material, further inquiries can be directed to the corresponding authors.

## Ethics Statement

The studies involving human participants were reviewed and approved by the Beijing Friendship Hospital, Capital Medical University. The patients/participants provided their written informed consent to participate in this study.

## Author Contributions

XQ, CD, XL, and NX collected the clinical and imaging data. XQ, PZ, and ZM performed the experiment. XQ drafted the manuscript. ZW, PZ, HD, and BG designed the study and ensured the questions related to all aspects of the work. ZY and SG gave critical comments on the manuscript. All authors contributed to the article and approved the submitted version.

## Conflict of Interest

The authors declare that the research was conducted in the absence of any commercial or financial relationships that could be construed as a potential conflict of interest.

## Publisher’s Note

All claims expressed in this article are solely those of the authors and do not necessarily represent those of their affiliated organizations, or those of the publisher, the editors and the reviewers. Any product that may be evaluated in this article, or claim that may be made by its manufacturer, is not guaranteed or endorsed by the publisher.
